# Single-cell mass cytometry in immunological skin diseases

**DOI:** 10.3389/fimmu.2024.1401102

**Published:** 2024-07-16

**Authors:** Mingming Zhao, Yuqi Cheng, Jinping Gao, Fusheng Zhou

**Affiliations:** ^1^ Department of Dermatology, The First Affiliated Hospital, Anhui Medical University, Hefei, Anhui, China; ^2^ Institute of Dermatology, Anhui Medical University, Hefei, Anhui, China; ^3^ Key Laboratory of Dermatology (Anhui Medical University), Ministry of Education, Hefei, Anhui, China; ^4^ Inflammation and Immune Mediated Diseases Laboratory of Anhui Province, Hefei, Anhui, China

**Keywords:** mass cytometry, imaging mass cytometry, proteome, autoimmune skin disease, multi-omics

## Abstract

Immune-related skin diseases represent a collective of dermatological disorders intricately linked to dysfunctional immune system processes. These conditions are primarily characterized by an immoderate activation of the immune system or deviant immune responses, involving diverse immune components including immune cells, antibodies, and inflammatory mediators. However, the precise molecular dysregulation underlying numerous individual cases of these diseases and unique subsets respond under disease conditions remains elusive. Comprehending the mechanisms and determinants governing the homeostasis and functionality of diseases could offer potential therapeutic opportunities for intervention. Mass cytometry enables precise and high-throughput quantitative measurement of proteins within individual cells by utilizing antibodies labeled with rare heavy metal isotopes. Imaging mass cytometry employs mass spectrometry to obtain spatial information on cell-to-cell interactions within tissue sections, simultaneously utilizing more than 40 markers. The application of single-cell mass cytometry presents a unique opportunity to conduct highly multiplexed analysis at the single-cell level, thereby revolutionizing our understanding of cell population heterogeneity and hierarchy, cellular states, multiplexed signaling pathways, proteolysis products, and mRNA transcripts specifically in the context of many autoimmune diseases. This information holds the potential to offer novel approaches for the diagnosis, prognostic assessment, and monitoring responses to treatment, thereby enriching our strategies in managing the respective conditions. This review summarizes the present-day utilization of single-cell mass cytometry in studying immune-related skin diseases, highlighting its advantages and limitations. This technique will become increasingly prevalent in conducting extensive investigations into these disorders, ultimately yielding significant contributions to their accurate diagnosis and efficacious therapeutic interventions.

## 1 Introduction

Single-cell analysis is pivotal for revealing structural and functional differences among individual cells. These techniques highlight significant cell-to-cell diversity, identify rare but functionally important subpopulations, and unveil unique characteristics of individual cells (1). Flow cytometry has been a powerful tool for single-cell analysis for an extended period (2). In recent years, with the advancement of high-throughput single-cell analysis methods, cellular subpopulation analysis has entered a new era of high dimensionality, surpassing the constraints of traditional methods for defining, characterizing, and quantifying immune cell subpopulations (3, 4). A variation of flow cytometry, known as cytometry by time-of-flight (CyTOF) or mass cytometry, was developed in 2009. Bandura et al. introduced the first purpose-designed prototype mass cytometer, showcasing its application in the simultaneous detection of 20 surface antigens within single cells of leukemia cell lines and leukemia patient samples (5). CyTOF is a single-cell proteomic analysis technique that assesses targeted intracellular and cell surface markers. It overcomes the limitations of traditional flow cytometry, which is often hindered by spectral overlap from fluorescent tags and less detected protein numbers. Instead, CyTOF employs antibodies conjugated to heavy metal isotopes, allowing simultaneous profiling of more than 50 markers. This allows detailed phenotypic and functional characterization of individual cells within a sample (6). Advances in CyTOF have led to the advent of imaging mass cytometry (IMC), a technique that combines metal-labeled antibody-based immunohistochemistry (IHC) with laser ablation and mass-spectrometry detection to produce multiplexed images, thus adding the tissue architectural information to CyTOF-based data (7). IMC has the capability to assess up to 40 protein markers on each cell in a single scan, utilizing 135 available detection channels. This surpasses the limitations of conventional IHC and immunofluorescence (IF) techniques, greatly enhancing the ability to explore intricate cellular systems and processes (8). IMC not only analyzes cells within their tissue context but also provides high resolution to precisely locate proteins within nuclear, cytoplasmic, and membranous cell compartments. In 2011, IMC technology was commercialized, leading to its gradual dissemination and extensive application (9). Presently, CyTOF/IMC has evolved into an indispensable method for investigating cellular heterogeneity in many autoimmune diseases, such as tumors, systemic lupus erythematosus (SLE), rheumatoid arthritis (RA) etc.

This paper presents an extensive review of the current applications of single-cell mass cytometry in immune-related skin diseases, including an overview of its working principles and primary functionalities. We emphasize the significance of this technique as a powerful tool for investigating skin immunology, with the potential to advance targeted therapies for various skin conditions.

## 2 Overview of working principles in single-cell mass cytometry

### 2.1 Mass cytometry

**Figure 1:**
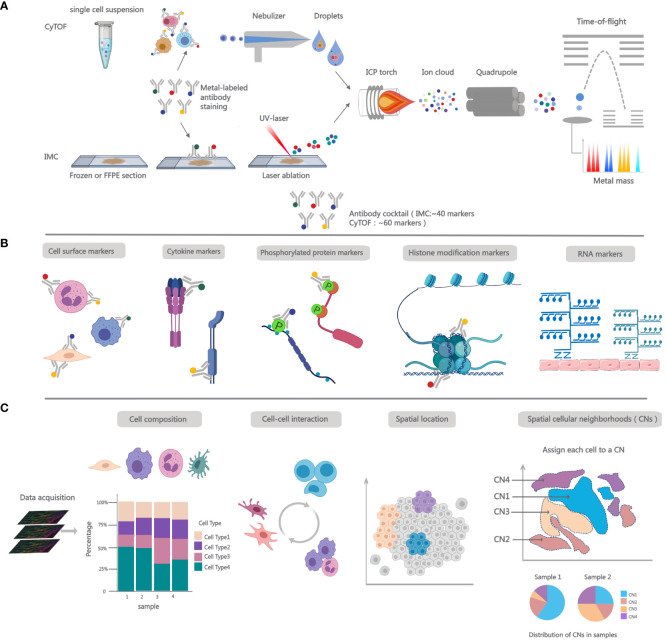
Overview of working principles and functions for single-cell mass cytometry. **(A)** Workflow for CyTOF and IMC. In CyTOF experiments, employ metal-conjugated antibodies to label cells and factors of interest in single-cell suspensions. After staining, cells are introduced into the nebulizer, where upon exit, they transform into fine droplets of spray, which are then carried to the ICP. There, they undergo ionization, forming an ion cloud. Low m/z ions are filtered using a quadrupole and concentrate heavy metal reporter ions to be quantified by time-of-flight mass spectrometer. In IMC analysis, Frozen or FFPE tissue sections are stained with metal-conjugated antibodies and then placed into the slide chamber. Tissue is ablated using UV laser, releasing metal isotopes bound to antibodies as ions. These ions are then carried to ICP using an inert gas flow. Subsequently, the isotopic content of each pixel is detected using a TOF mass spectrometer. **(B)** Single-cell mass cytometry measurement breadth. More than 40 metal-conjugated antibodies are formulated into antibody cocktails, which can be utilized for cell phenotyping and the quantification of intracellular cytokines; adding antibodies targeting phosphorylated proteins to the panel allows for the assessment of cell signaling; the panel can also incorporate markers for histone modifications or probes for detecting mRNA transcripts. **(C)** The robust functionalities of IMC. After image acqusition, the data can be employed for immune phenotyping to ascertain the proportions of cell populations, spatial interactions among cells, and spatial anatomical locations. In addition to pairwise interactions, spatial cellular neighborhoods (CNs) can aid in assessing multicellular structural interactions within tissues. The count of interacting cells within a neighborhood (N) and the total number of cellular neighborhoods vary across different samples.

CyTOF is essentially an inductively coupled plasma mass spectrometry (ICP-MS) with a time-of-flight (TOF) detector, created for precise single-cell measurements or bead-based immune detection assays. It provides a wide mass detection range, including nearly 100 mass detection channels (10, 11). In CyTOF, antibodies are labeled with stable heavy metal isotopes and bind specifically to cell surface and intracellular markers, functioning similarly to traditional flow cytometry’s fluorescent labels. After cell staining, cells are introduced into the CyTOF analyzer, where they are nebulized into droplets. Subsequently, droplets enter the inductively coupled argon plasma (ICP), where they undergo evaporation, atomization, and ionization (12, 13). The ion cloud is filtered through a quadrupole to eliminate common biological elements and then analyzed using a TOF mass spectrometer. The signal intensity for each isotope correlates with specific antibodies, enabling the measurement of analyte levels within the cell (14). (**Figure 1A**) For mass cytometry data analysis, the t-distributed stochastic neighbor embedding (t-SNE) algorithm (15), Spanning-tree Progression Analysis of Density-normalized Events (SPADE) (16, 17), FlowCore (18) and CATALYST can be used to visualize high-dimensional data (19). FlowSOM (20), X-Shift (21) and PhenoGraph (22) are employed to identify cell populations. Cellular development and lineage decisions can be tracked using trajectory algorithms, which order cells based on gradual phenotypic changes.

### 2.2 Imaging mass cytometry

IMC extends the multiplexed analysis capabilities of CyTOF-based mass cytometry to enable spatially resolved measurements (16, 23). The IMC workflow integrates mass cytometry, ICC, and IHC analyses with a high-resolution laser ablation system, enabling the examination of adherent cells and tissue sections at a remarkable cellular resolution of 1µm. IMC processing follows the same pre-treatment protocol as IHC/IF, involving specimen preparation, antibody incubation, and subsequent washing before analyzing antibody signals in cell/tissue specimens (7). The next step is passing the stained histology slide through an ICP ion source equipped with a high-energy UV laser for tissue ablation. The pixels are ablated to create ion clouds with heavy metals, then sorted by mass-to-charge ratio (m/z) and quantified using a TOF mass spectrometer. (**Figure 1A**).

## 3 Unraveling the complexity of immune landscape with single−cell mass cytometry

The intricate makeup of human immune cell populations, their diverse functional statuses, and their various locations necessitates the use of appropriate methods for a thorough evaluation (24). Single-cell RNA sequencing (scRNA-seq) allows for the comprehensive analysis of the entire transcriptome in thousands of individual cells (25). Cellular indexing of transcriptomes and epitopes by sequencing (CITE-seq) uses oligonucleotide-conjugated antibodies to enable the simultaneous measurement of RNA and surface proteins (26). The evolution of these technologies has made it feasible to dissect individual cells at multiple omics levels, contributing to a comprehensive exploration of the composition, activation, and their relevance to diseases of immune cells.

While mRNA sequence dictates the amino acid sequence of the produced polypeptide, its abundance does not precisely reflect protein translation levels (27). As pivotal biomolecules governing fundamental biological processes, proteins undergo post-translational modifications that regulate their stability and functionality, thereby influencing cellular phenotypes and biological activities (28). The investigation into proteins has been facilitated by the development of single-cell proteomic technologies, such as mass cytometry. This novel platform enhances signal resolution, increasing the number of parameters that can be simultaneously measured by at least tenfold. It is particularly well-suited for multi-parameter analyses of diverse biological samples, such as tumors (29). Mass cytometry can be employed to analyze the phenotypic characteristics of various cells of interest (30), including rare functional immune cell subpopulations (31). It enables the exploration of how different cell subpopulations interact and identifies those that are functionally dysregulated, providing crucial insights into understanding the pathogenic mechanisms of many autoimmune diseases (32, 33). CyTOF has been successfully integrated with analyses of histone modification patterns and epigenetic codes, allowing for a deeper understanding of the complex biology of target cells (34). Additionally, utilizing metal-conjugated antibodies for phosphorylated proteins, CyTOF enables probing intracellular signal transduction in individual cells (23), covering apoptosis, metabolism, proliferation, and activation states of signaling pathways (35–38) (**Figure 1B**). This aids in the discovery of novel pathways for drug development.

Many single-cell studies, including CyTOF, lack spatial context and rely on isolated tissues, potentially leading to the loss of some cell types and proteins, while overlooking the importance of the extracellular matrix (39). Multiplexed Ion Beam Imaging (MIBI) (40) and IMC rely on mass spectrometry to detect metal isotopes coupled to antibodies, differing by their use of an ion beam or laser, respectively, for tag ionization. Additionally, tissue-based cyclic immunofluorescence (t-CyCIF) (41) and oligonucleotide-tagged antibody technology CODEX (42) have also been employed to provide multi-parameter epitope quantification with an imaging context. These emerging technologies have significantly advanced the exploration of detailed spatial and functional maps of complex tissues. Spatial analysis can offer a glimpse into the microenvironment of cells, unveiling intricate cell-cell interactions (**Figure 1C**). This is crucial for assessing cellular characteristics and functional states within the biological environment (43). The effectiveness of anti-tumor immunity hinges upon cellular interactions within the tumor microenvironment (TME) (44, 45). Researchers utilized IMC for protein imaging in human Formalin-Fixed, Paraffin-Embedded (FFPE) breast cancer samples. They observed heightened phosphorylation of the S6 protein in stromal cells, often situated near the tumor periphery, suggesting that signaling activity in stromal cells might be induced by tumor cells (7). IMC has also been employed to explore the TME of melanoma. Interestingly, in pre-treatment melanoma, the abundance of proliferating antigen-experienced cytotoxic T cells (CD8+ CD45RO+ Ki67+) and the proximity of antigen-experienced cytotoxic T cells to melanoma cells correlated with positive responses to immune checkpoint inhibitors (ICIs) (46) (**Figure 1C**). Beyond proteins, IMC offers the capability for spatial visualization of mRNA. This technique, called RNAscope, facilitates the identification of biomarkers with low expression levels or localized concentration, such as secreted factors including cytokines and chemokines (47) (**Figure 1B**). A recent investigation employed IMC-based concurrent detection of mRNA and proteins to spatially profile the chemokine landscape in FFPE samples from metastatic cutaneous melanoma (48).

Prior studies highlight the substantial potential of IMC in revealing cellular spatial organization, pinpointing rare cellular subpopulations, characterizing cell morphology, elucidating cell proliferation, and unraveling gene expression dynamics (49). By employing IMC to investigate the heterogeneity and functionality of autoimmune diseases, we anticipate that this will propel medicine toward personalized molecular-targeted diagnostics and treatments.

## 4 Application of single-cell mass cytometry in immunological skin diseases

Numerous skin diseases are intricately associated with immune system dysregulation, such as systemic lupus erythematosus, scleroderma, dermatomyositis, vitiligo, psoriasis, pemphigus, and others. The precise etiology and pathogenic mechanisms of many autoimmune skin disorders remain unclear. This presents a significant impediment to the development of targeted therapeutic agents and the effective treatment of affected individuals (50). Unraveling the immunological mechanisms underlying these conditions has become a focal and formidable challenge in contemporary scientific research.

The complexity of immune infiltrates and the unique functions of specific subgroups in both normal and pathological states have proven inscrutable with conventional methodologies. This challenge is particularly pronounced when different subpopulations express similar markers (51). Commencing in 2014, several collaborative initiatives have been launched, such as the Accelerating Medicines Partnership (AMP) for Rheumatoid Arthritis and Systemic Lupus Erythematosus networks, with the aim of identifying novel therapeutic targets for autoimmune diseases RA and SLE. Leading-edge technologies such as CyTOF, scRNA-Seq and ATAC-Seq are currently employed in the exploration of many autoimmune diseases’ underlying mechanisms. These single-cell investigations have represented a pivotal milestone, offering fresh perspectives on the diversity of immune cells in the context of diseases. CyTOF has the potential to facilitate a deeper understanding of the heterogeneity, development, hierarchy, and interplay of cutaneous cellular phenotypes with other tissues (52, 53). Additionally, CyTOF can be employed for investigating post translational protein modifications, such as protein phosphorylation involved in cellular signaling, thereby effectively addressing a limitation of scRNA-seq (34, 54). IMC extends the capabilities of CyTOF technology, it presents an additional and substantial advantage by enabling the resolution of cellular spatial orientation within tissues. For many autoimmune skin diseases, the utilization of tissue-based imaging methods holds significant relevance in investigating immune cells and stromal cells within their natural or pathological contexts. IMC empowers the generation of high-resolution, multi-layer images that illustrate protein expression, cellular localization, and *in situ* interactions, thus enhancing its utility in dermatology research (55).

In this segment, we encapsulate the current investigations into the application of CyTOF/IMC in immunological skin conditions, aiming to gain deeper insights into the pathogenic mechanisms linked to the diversity of immune cells within these diseases (**Tables 1**, **2**).

**Table 1:** Overview of studies in immunological skin diseases using CyTOF.

No	Disease Types	Targeted cells	Sample types	Sample size	Discoveries by CyTOF	Clinical relevance	Reference
1	PSO, AD	Dendritic cells, macrophages	Skin, PBMC	PSO (n =21), AD (n=15)	CD14+ DC3s increased in PSO and co-produced IL1B and IL23A.	Drugs targeting IL23, such as guselkumab are in clinical use for PSO.	(56)
2	PSO	Immune cells	Skin	PSO (n=20), HCs(n=15)	The epidermal immune microenvironment played a dominant role in PSO.	Not mentioned.	(57)
3	PSO	T cells	PBMC	PSO (n =38), HCs(n=30)	The CD3-CD4+cells had elevated OX40 and decreased FRA2.	KHK4083(anti-OX40) has been in a Phase I clinical trial for PSO.	(58)
4	PSO	Immune cells	PBMC	PSO (n=32), HCs(n=10)	Intracellular pp38 and pERK in Th cells were associated with disease severity.	pp38 and pERK correlate with disease severity.	(59)
5	PSO	Tregs	PBMC	PSO (n=31), HCs(n=32)	Psoriatic circulating Tregs exhibited an impaired skin-trafficking phenotype.	The frequency of Tregs correlate with PASI scores.	(60)
6	PSO, PsA	Immune cells	PBMC	PSO (n=12), PsA (n=7)	CD28 and CD127, specifically differentiated PsA from PsV.	Abatacept(blocking CD28 interaction on T cells)is in clinical trials for PsA.	(61)
7	PsA, RA	T cells	SFMC	PsA (n =7), RA (n=5)	Type17 CD8+ T cells enriched in the PsA joint.	IL-17 inhibitors, such as secukinumab, ixekizumab, brodalumab and bimekizumab are approved for treating PsA.	(62)
8	PsA	Leukocytes	SFMC, PBMC	PsA (n=10)	CXCR3 upregulated in synovial CD8 T cells, while CXCL9 and CXCL10 elevated in PsA SF.	Elevated CXCL10 is known to predict the future development of PsA in patients with PsC.	(63)
9	PsA, RA	Immune cells	PBMC	PsA (n =27), RA (n=14)	pSTAT3 levels increased in all the CD4+ T cell subsets analyzed.	TYK2 inhibitors like deucravacitinib have shown efficacy in PsA.	(64)
10	PsA, RA	Leukocytes	PBMC	PsA (n =16), RA (n=21), HCs(n=13)	CD8+ T cells, B cells, MAIT/iNKT, and ILCs elevated in PsA.	Not mentioned.	(65)
11	PsA	Leukocytes	SFMC, PBMC	PsA(n=11), HCs(n=15)	Monocytes and macrophages produced osteopontin and CCL2 in PsA SF.	Osteopontin could be a clinical biomarker for PsA.	(66)
12	PSO, AD	T cells	PBMC	PSO (n=19), AD (n=15), HCs(n=9)	Skin-homing TRcM cells with IL-22 production were elevated in AD.	Fezakinumab (anti-IL-22) has showned efficacy in a clinical trial.	(67)
13	AD	T cells	PBMC	AD(n=20), HCs(n=15)	IL-21 expression in IL-13+ T cells correlated with AD severity	JAK1 inhibitors like updacitinib can inhibit IL-21R signaling, showing efficacy in AD.	(68)
14	SLE	Immune cells	PBMC	SLE (n =8), HCs(n=17)	CD14hi monocytes showed a unique signature of MCP1 in SLE patients.	MCP1 neutralization may serve as an anti-inflammatory therapy for SLE.	(69)
15	SLE	Monocytes	PBMC	cSLE (n=10), HCs(n=10)	Inhibiting JAK eliminates the cytokine profile induced by cSLE plasma in CD14hi monocytes.	JAK inhibitor baricitinib showed positive results in a phase 2 study.	(70)
16	SLE	Monocytes	PBMC		The U1-snRNP stimulated MIF production in monocytes, which, in turn, regulated NLRP3 activation and IL-1β production.	Milatuzumab (anti-CD74 antibody against MIF) is in clinical trials for SLE.	(71)
17	SLE	Immune cells	PBMC	ANA+healthy (n=24), ANA-healthy (n=24), SLE (n=24)	ANA+ healthy individuals with lower lupus risk displayed a unique immune endotype.	Not mentioned.	(72)
18	SLE	Immune cells	PBMC	SLE (n=28), HCs(n=15)	SLE ICPs can categorize into five clusters based on the proportion of Ki-67.	Assessing ICP proliferative activity can be correlated with the clinical phenotypes of SLE.	(73)
19	SLE	B cells	PBMC	SLE (n=30), HCs(n=30)	B cell populations in SLE increased with an activated phenotype, lacking CD21 and CD27.	CD21−CD27− B cells can be a biomarker for assessing SLE disease activity.	(74)
20	SLE	T cells	PBMC	SLE (n=22), HCs(n=8)	CD8+CD27+CXCR3− T cells were increased in rSLE compare to aSLE.	CD8+CD27+CXCR3− T cells may serve as key biomarkers for SLE remission.	(75)
21	SLE	Leukocytes	PBMC	SLE (n=20), HCs(n=20)	CD38 increased on SLE immune cells.	Mezagitamab (anti-CD38) has shown effectiveness in treating SLE.	(76)
22	SLE	PBMCs	PBMC	Not taking MMF(n=10), Taking (n=5)	MMF reduced STAT3 phosphorylation in SLE patients.	MMF has shown effectiveness in treating SLE.	(77)
23	SLE	PBMC	B cells	SLE (n=79), HCs(n=80)	CXCR5–CD19^low^ B cells, which are precursors of plasmablasts, increased in SLE.	Belimumab targets early, transitional B cells and partially targets PBs/plasma cells.	(78)
24	SSc	Immune cells	PBMC	SSc (n =20), HCs(n=10)	The study revealed disease-induced peculiarities in the immune architecture.	Not mentioned.	(79)
25	SSc, SLE, pSS	Immune cells	PBMC	SADs (n =169), HCs(n=44)	All autoimmune diseases displayed diverse frequencies of immune cell subsets.	Not mentioned.	(80)
26	SSc	T cells	PBMC	SSc (n =17), HCs(n=9)	A new CCR5+CD28+ DNT cell subset decreased in SSc.	CCR5+CD28+ DNT subset may indicate disease activity in SSc.	(81)
27	HS	Immune cells	PBMC	HS (n=18), HCs(n=11)	Memory B cells, plasmablasts, late NK cells, pDCs and I.Monos exhibited increased CD38 in HS.	Anti-CD38 immunotherapy may be a new management strategy for HS.	(82)
28	TEN	T cells	Skin, Blisters, PBMC	TEN(n=18), MPE(n=14), HCs	Effector memory polycytotoxic CD8+ T cells are the main leucocytes in TEN blisters at the acute phase.	Not mentioned.	(83)
29	JDM	Immune cells	PBMC	JDM (n=17), HCs(n=17)	PLCγ2 phosphorylation dysregulated in NK cell.	Not mentioned.	(84)
30	DM, PM	T, B cells	PBMC	DM or PM (n=12)	Positive correlations were observed between the CD4/CD8 ratio in DM at CTLA4‐Ig treatment.	CTLA4-lg (abatacept) has showned efficacy in treating DM.	(85)

PBMC, Peripheral Blood Mononuclear Cells; SLE, systemic lupus erythematosus; cSLE, childhood-onset systemic lupus erythematosus; PSO, psoriasis; AD, atopic dermatitis; PsA, psoriatic arthritis; SFMC, Synovial Fluid Mononuclear Cells; SF, Synovial Fluid; RA, rheumatoid arthritis; SSc, systemic sclerosis; pSS, primary sjögrens syndrome; SADs, systemic autoimmune diseases; HS, hidradenitis suppurativa; TEN, toxic epidermal necrolysis; MPE, maculopapular exanthema; JDM, juvenile dermatomyositis; DM, dermatomyositis; PM, polymyositis.

**Table 2:** Overview of studies in immunological skin diseases using IMC.

No	Disease Types	Target cells	Sample size	Discoveries by IMC	Clinical relevance	Reference
1	DM	Immune cells	DM(n=10), HCs (n=5)	IFNβ is abundant in all cells present in DM skin.	The IFN–chemokine score correlates with disease activity.	(86)
2	DM, ASyS	Immune cells	DM (n=7), ASyS (n=5)	The Type I interferon system played a role in the DM-like skin lesions.	The IFN–chemokine score correlates with disease activity.	(87)
3	DM, cSLE	Immune cells	DM (n=6), cSLE (n=4)	JDM skin showed a strong innate immune signature and endothelial –immune cell interaction.	Not mentioned.	(88)
4	DM	Immune cells	DM(n=10), HCs (n=5)	CB2R was upregulated on immune cells, especially DCs in DM.	Lenabasum (CB2R agonist) is being investigated as a potential treatment for DM.	(89)
5	CLE	Myeloid cells	CLE (n=7), HCs (n=14)	Lupus-enriched CD16+DCs underwent IFN education in the skin.	Sifalimumab (anti-IFN-α) and anifrolumab (IFN receptor-blocking) have been evaluated in clinical trials for SLE.	(90)
8	CLE	Immune cells	SCLE(n=19), DLE(n=19)	pDCs were not the major producers of IFN-1 in CLE.	Litifilimab and VIB7734 (targeting pDCs) are under investigation for CLE.	(91)
6	CLE	B cells	CLE(n=50), HCs (n=5)	DLE lesions harbored a enrichment of B cells compared to ACLE and SCLE.	The skin B cell score could be a clinical marker for SLE risk assessment.	(92)
7	CLE	Immune cells	CLE (n=44)	The immune cell composition differed among CLE patients.	Categorizing patients by their immune cell composition in CLE enables the stratification of drug responses.	(93)
9	SSc	Immune cells	SSc(n=5), HCs (n=5)	SSc skin exhibited elevated IL1RAP and upregulated IL1RAP-associated signaling molecules.	The anti-IL1RAP antibody CAN10 has entered a phase 1 clinical trial.	(94)
10	SSc	Endothe-lial cells and immune cells.	SSc (n=19), HCs (n=14)	CD34+αSMA+CD31+ VECs as a novel EC population was increased in SSc.	CD34+αSMA+CD31+ VEC counts correlate with fibrotic remodeling outcomes.	(95)
11	HS	Plasma cells and B cells	HS (n=22), HCs (n=10)	Plasma cells and B cells are key infiltrating leukocytes in HS, with BTK and SYK pathways central to signaling.	BTK inhibitors and SYK inhibitors could be new management strategies for HS.	(96)
12	MDR	T cells	COVID-MDR (n=4), MDR (n=7) and DRESS (n=4)	A systemic cytokine storm might promote activation of Mo/Mac and cytotoxic CD8+ T cells.	Not mentioned.	(97)

CLE, cutaneous lupus erythematosus; DLE, discoid lupus erythematosus; ACLE, acute cutaneous lupus erythematosus; SCLE, subacute cutaneous lupus erythematosus; ASyS, antisynthetase syndrome; MDR, maculopapular drug rashes; DRESS, drug rash with eosinophilia and systemic symptoms.

### 4.1 Mass cytometry

#### 4.1.1 Psoriasis (PSO)

Psoriasis is a chronic, autoimmune skin disease that affects approximately 125 million people worldwide. Psoriasis has various clinical phenotypes, but the most frequent is chronic plaque or psoriasis vulgaris. It is chacterized by red plaques covered with silvery scales, accompanied by itching and discomfort (98). These skin lesions typically manifest on the elbows, knees, scalp, and sacral region, and can also involve nails and joints. Psoriatic arthritis, a commonly occurring comorbidity, manifests in approximately 30% of individuals with psoriasis and exhibits a wide range of clinical features, often leading to a delay in diagnosis and treatment (99). The immune mechanism leading to tissue damage in psoriatic arthritis remains elusive. Applying CyTOF to PSO/PsA blood, skin tissue, synovial fluid (SF) and synovial tissue can help us understand the cellular landscape, identify new biomarkers, and uncover therapeutic targets.

Inflammatory skin diseases, including PSO and AD are underpinned by DC–mediated T cell responses. The heterogeneous human cutaneous DC population is not yet fully characterized, and its contribution to these diseases remains unclear (100). Xue et al. documented two DCs (cDC2), while Cytlak et al. disclosed the existence of CD141+cDC1, CD1c+CD14-DC2, and CD1c+CD14+ DC3 in both human blood and dermis (101). These findings imply the presence of DC3 in human skin, however, the precise contribution of DC3 in psoriasis pathogenesis remains ambiguous. Based on CyTOF data, CD14+ DC3s were found to be increased in PSO lesional skin, co-producing IL1B and IL23A, which are pathological in PSO (56). IL-23 and Th17 responses are considered important drivers of psoriasis (102, 103). IL23 can promote the differentiation and activation of Th17 cell. Drugs targeting IL23, such as ustekinumab, guselkumab, and risankizumab, are currently in clinical use for psoriasis (104, 105). Hence, it might possible to discover new therapeutic targets for PSO by developing drugs that inhibit metabolism by targeting IL1B/IL23A co-producing CD14+ DC3s. Subsequently, Zhou et al. (57) used CyTOF to analyze DC subsets in psoriatic epidermis and dermis. They found that CD1c+CD11b+cDC2s migrated to the epidermis in pre-lesional skin, replacing EpCAM+CD11c^low^ LCs and triggering inflammation. CD207+CD11c^hi^ LCs and CD5+ T cells also accumulated in the epidermis, driving psoriasis inflammation. The epidermal immune environment was more significant than the dermal one, aligning with psoriasis inflammation.

PSO is characterized by the presence of activated T cell subtypes in the skin, which secrete proinflammatory cytokines. This T cell-mediated immune imbalance is at the core of the pathogenesis of this widespread inflammatory skin disease. In 2019, researchers used CyTOF and IMC technologies to analyze immune cells in peripheral blood and lesional skin. The study identified three novel subsets abundant in the peripheral blood of PSO patients, resembling CD3–CD4+ lymphoid tissue inducer cells, Tc17 cells, and CD8+CXCR3+ Tregs. The CD3–CD4+ cells had elevated OX40 and reduced FRA2 expression, with these markers positively correlating with the PS area and severity index (PASI) (58). Notably, OX40-OX40L, as an immune checkpoint, has been proposed as a potential therapeutic target to treat psoriasis. An anti-OX40 monoclonal antibody (KHK4083) has been administered to patients with PSO in a phase I clinical trial, showing improved outcomes (106). Furthermore, mass cytometry analysis of peripheral blood before and after biologic therapy revealed an increase in circulating Th17, Th22, Th9, and cytotoxic T cells in severe psoriasis. The intracellular pp38 and pERK in T helper cells correlated with disease severity (59).

Accumulating evidence has shown an important role of tissue-resident memory T (T_RM_) cells in the pathogenesis of psoriasis (107). A recent study used CyTOF and scRNA-seq to analyze SF CD8+ and CD4+ CD69+CD103+ T_RM_ cells in PsA and RA patients. Three distinct CD8+ CD69+CD103+ T_RM_ cell populations were identified within inflamed arthritic joints: cytotoxic and Treg-like T_RM_ cells, found in the synovial joint of both PsA and RA patients, while type 17-like T_RM_ cells and type 17-like CD8+CD103 T cells were specifically enriched in PsA patients (62). These data shed light on a potential underlying reason for the differential clinical efficacy of secukinumab, an IL-17A blocker, in treating PsA compared to RA (108): a significantly larger fraction of IL-17A-secreting tissue-resident and non-resident CD8+ T cells within the synovial PsA joint may contribute to the immunopathology and persistence of this disease. Exploration of T_RM_ cells diversity persists. Using mass cytometry, a study revealed a three-fold increase in memory CD8+ T cells in the synovial fluid compared to peripheral blood in PsA patients (63). These cells express cell-cycle activation, tissue-homing and tissue residency markers, including the skin or gut-homing marker ITGA1 and granulysin. Remarkably, CXCR3 was upregulated in the expanded synovial CD8+ T cells, and its two ligands, CXCL9 and CXCL10, were elevated in PsA SF. Elevated CXCL10 is known to predict the future development of PsA in patients with cutaneous-only psoriasis (PsC) (109).

Studies have shown that overexpression of STAT3C, an active form of STAT3, in CD4+ T cells induces many major features of PsA in an animal model (110, 111). Recent CyTOF research examined pSTAT3 levels in circulating immune cells from PsA patients during active and inactive phases. The results showed heightened STAT3 signaling in CD4+ T cells (especially Th1 and Tfh subtypes) and CD14+CD16-monocytes during active PsA (64). Increased STAT3 expression in these cells suggests their recruitment to inflammation sites and underscores their role in PsA immunopathology. The effectiveness of TYK2 inhibitors like deucravacitinib supports STAT3’s pivotal role in PsA (112). In a later study, scientists used CyTOF comprehensively analyzed 47 immune cell subpopulations in the peripheral blood of active PsA patients, comparing them to healthy controls (HCs) and active RA patients, both seropositive and seronegative. The analysis showed higher frequencies of naïve and activated CD8+ T cells, B cells, MAIT/iNKT, and ILCs in PsA compared to seropositive RA, while the opposite was observed for terminal effector, senescent, and Th2-like cells (65).

Past research, often centered on specific immune cell types in PsA SF, has emphasized the potential roles of CD8 T cells (113). However, the significance of other myeloid populations in PsA has been comparatively underexplored. By utilizing CyTOF, Nicole Yager et al. observed pronounced shifts in the myeloid component of PsA SF when compared to blood samples. This included an expansion of intermediate monocytes, macrophages, and dendritic cell populations. Importantly, these myeloid cells exhibited substantial production of osteopontin and CCL2, even in the absence of *in vitro* stimulation (66). Studies have shown that osteopontin serum and SF levels correlate with C-reactive protein in RA patients (114, 115). Here, they found that elevated serum osteopontin was associated with PsA, indicating it as a potential clinical biomarker for PsA. Additionally, CCL2 inhibition has shown efficacy in rat adjuvant arthritis (116). Future research should investigate the potential of CCL2 inhibition in PsA treatment.

#### 4.1.2 Atopic dermatitis (AD)

AD is the most common chronic inflammatory skin disease which often develops during childhood. It is characterized by recurrent eczematous lesions and intense itch and discomfort. The pathophysiology of AD entails a complex interplay of factors, including a significant genetic predisposition, impaired functioning of the epidermis, and the initiation of inflammation driven by T-cells (117, 118). While whole tissue biopsy studies and analysis of blood composition have contributed to a better understanding of AD, the specific molecular alterations at the single-cell level remain largely unexplored.

AD is linked to the activation of various T-cell subsets. While it’s widely recognized for robust Th2 immune responses, other pathways like Th22, Th17/IL-23, and Th1 cytokines may also contribute, especially in certain AD subtypes (119, 120). Therefore, it is crucial to thoroughly investigate the circulatory T-cell phenotype in AD. In a prior CyTOF study, T-cell subsets within healthy skin were investigated (121). Nevertheless, AD lacked a comprehensive mass cytometry analysis until recent limited data emerged, by using CyTOF, researchers provided an exhaustive description of the circulatory T-cell compartment, highlighting phenotypic and functional differences in patients with AD and patients with psoriasis (122). Particularly, they identified recirculating memory T (TRcM) cells by the expression of CD103. Skin-homing TRcM cells were the major IL-22 producers within CD8+T cells, with patients with AD producing significantly more IL-22 than HCs. IL-22 production in TRcM cells was correlated with AD disease severity. Moreover, a clinical trial has demonstrated the efficacy of blocking IL-22 with fezakinumab in treating moderate-to-severe AD patients (123). IL-22–producing skin-homing TRcM cells could serve as promising candidates for future studies to identify biomarkers of response to IL-22 blockade in AD patients.

In order to further characterize the immune profile of AD, Czarnowicki et al. implemented a surface, cytokine, and transcription multi-biomarker CyTOF panel to investigate T cell polarization in the blood of AD patients versus controls (68). They discovered a correlation between IL-21 expression in IL-13+ T cells and AD severity, suggesting a potential role for IL-21 in AD. It is noteworthy that IL-21 signals through the JAK/STAT pathway, and JAK1 inhibitors, such as abrocitinib, baritinib, and updacitinib, have recently been demonstrated to block IL-21R signaling, showing efficacy in AD (124, 125). Further studies are needed to precisely determine the contribution of IL-21 to AD pathogenesis.

#### 4.1.3 Systemic lupus erythematosus (SLE)

SLE stands as a complex autoimmune disorder characterized by multifaceted organ involvement and perturbed immune system responses. SLE is characterized by a wide range of clinical manifestations including fatigue, fever, joint pain, joint swelling, and skin rash etc., which leading to an unpredictable disease trajectory. Advancements in research have revealed that the pathogenesis of SLE involves aberrant production and activation of various immune cells, inflammatory mediators, and immune complexes (126, 127).

CyTOF has been employed by many researchers to investigate the heterogeneity of immune cells in peripheral blood of SLE patients, greatly enhancing our understanding of the functional and status changes within various immune cell subsets as a consequence of the aberrant immune responses in SLE disease state.

In 2015, researchers stimulated peripheral blood samples with TLR ligands while concurrently performing mass cytometry analysis of surface marker expression, intracellular signaling protein activation status, and cytokine production (69). CD14hi monocytes exhibited the most polyfunctional cytokine expression patterns. In SLE patients, these monocytes showed a unique signature of MCP1, Mip1β, and TNFα, with MCP1 being the most prominent. It’s worth noting that MCP1 can recruit monocytes and lymphocytes to sites of inflammation and its elevated levels are linked to disease activity in various autoimmune conditions (128–130). Study has demonstrated that MCP1 neutralization can ameliorate disease symptoms in rodent models of SLE (131). Neutralizing MCP1 may serve as an adjunct anti-inflammatory therapy for SLE. In subsequent research, scientists found that exposure to cSLE plasma induced a unique cytokine signature in CD14hi monocytes (elevated MCP1, Mip1β, and IL-1RA) in the blood of HCs, which could be abrogated by selectively inhibiting JAK1/JAK2 signaling (70). Type I interferons, TLRs, and other pro-inflammatory cytokines involved in the pathogenesis of SLE transmit signals through the JAK/STAT signaling pathway, thereby regulating critical cellular functions such as survival, proliferation, and differentiation (132–135). Recently, JAK inhibitors have been incorporated into treatment regimens for autoimmune diseases. The JAK1 and JAK2 inhibitor baricitinib showed positive results in a Phase 2 study in SLE patients (136).

The exploration of SLE continues. Experimental evidence implicates the Macrophage Migration Inhibitory Factor (MIF) and the NLRP3 inflammasome in SLE pathogenesis and progression (137–139), but their precise molecular interplay remains unclear. Shin et al. used CyTOF to study monocyte phenotypes associated with MIF-related molecules and their changes after snRNP immune complex stimulation. They found that the U1-snRNP immune complex specifically stimulates MIF production, which plays an upstream role in regulating NLRP3 inflammasome activation and IL-1β production in activated monocytes (71). Study has shown that MIF antagonism reduces both the functional and histological indices of glomerulonephritis, as well as the expression of inflammatory cytokines and chemokines, in lupus-prone MRL/lpr or NZB/NZW F1 mice (140). These findings support the therapeutic potential of targeting MIF-dependent pathways in SLE, currently being investigated in clinical trials using anti-CD74 (141).

SLE is influenced by environmental and genetic factors. A recent study explored SLE heterogeneity at the single-cell level in ANA+ healthy individuals from diverse racial backgrounds. They identified a unique immune signature in ANA+ European Americans, characterized by a suppressive immune phenotype and reduced CD11C+ autoimmunity-associated B cells. This signature was absent in SLE patients, ANA- individuals, and African Americans, suggesting it may protect against disease transition (72).

Many peripheral immune cell populations (ICPs) change with disease progression, but their association with SLE clinical phenotypes remains unclear. Akiko Kajihara et al. used mass cytometry to analyze PBMCs from SLE patients, identifying 30 ICPs. By measuring the expression of specific markers and Ki-67 in CD45+ cells, they classified SLE patients into five clusters with distinct phenotypes (73). Horisberger et al. employed mass cytometry to investigate peripheral B cells from 30 SLE patients and 30 HCs. They identified CD21−CD27− B cells as a reliable biomarker for assessing SLE disease activity (74). In addition, researchers using mass cytometry to study immune cell dysregulation in peripheral blood samples from active SLE (aSLE), remission SLE (rSLE), and HCs found that the abundance and dysfunction of CD8+CD27+CXCR3−T cells could be potential biomarkers for SLE prognosis and concomitant diagnosis (75).

With the development of high-dimensional CyTOF technology, SLE classification is poised to shift from clinical to molecular phenotypes. This transition is expected to streamline the identification of patients suitable for specific targeted therapies.

### 4.2 Imaging mass cytometry

#### 4.2.1 Dermatomyositis (DM)

Idiopathic inflammatory myopathies (IIM) encompass a group of inflammatory muscle disorders characterized by varying degrees of involvement of additional organs. Among adults, DM and polymyositis (PM) are prevalent forms of IIM, while juvenile DM is the most commonly observed IIM in children (142). DM pathogenesis involves an immune-driven process triggered by environmental factors in genetically susceptible individuals (143).

Cutaneous inflammation has been shown to be associated with systemic disease activity and chronicity in DM (144). However, our understanding of the underlying mechanisms and immune cells driving cutaneous inflammation and disease-specific characteristics remains limited. Conducting an in-depth analysis of the inflammatory infiltrate in DM holds significant importance.

A recent study employing IMC to phenotype adult DM skin identified 13 distinct immune cell populations, predominantly myeloid, including abundant CD14+ macrophages and CD11c+ myeloid dendritic cells (mDCs), as well as lymphoid cells. The CD14+ monocyte/macrophage population correlated with disease activity. The study also found IFNβ protein was highly upregulated in the T cell, macrophage, DC, and endothelial cell populations of DM skin (86). In a subsequent study has once again highlighted the involvement of the type I IFN system in skin lesions of DM. Pathways associated with the type I IFN system, such as pSTING, pIRF-3, IFNβ, IFNκ, IFNα, IRF-5, TYK2, and TBK1, exhibited increased activity in both DM and Antisynthetase syndrome(ASyS). Interestingly, researchers revealed a potential divergence in pSTING+ macrophage pathways between ASyS and DM (87). The pathogenesis of DM is attributed to the activation of the type I interferon system, specifically IFNβ (145, 146). Upregulation of genes associated with type I interferons, cytokines (such as IL-6 and IL-1), and chemokines (including CXCL10, CXCL9, and CXCL11) can damage skin and muscle tissues (147). The IFN–chemokine score correlates with disease activity in both adult and juvenile DM cohorts (148). Investigating the origins and effects of type I interferons is crucial for identifying therapeutic targets to regulate disease activity (149).

In a study by Jessica et al., IMC was employed to characterize inflammatory cell populations and cell–cell interactions within juvenile DM lesional skin versus the childhood-onset systemic lupus erythematosus (cSLE). They found differences in cell populations, such as CD14+ macrophages, pDCs, and CD8+ T cells, in juvenile DM versus cSLE, and highlight a predominance of innate immune cells and endothelial cell–immune cell interactions in juvenile DM skin (88). IMC was also used to explore potential treatment targets for DM with Lenabasum, a cannabinoid type 2 receptor (CB2R) agonist. CB2R upregulated on immune cells in skin and blood, and in particular DCs. Demonstrating CB2R expression on key immune cells and its pathway effects can better guide this therapy in identifying DM patients with susceptible lesions (89).

Research has demonstrated the significance of IMC technique in the analysis of subcellular populations and identification of single-cell protein expression within the context of DM skin tissue microenvironment. Work using IFN signaling as a biomarker and using IMC findings as a rational basis for treatment and therapy will be a challenge for DM researchers in the future.

#### 4.2.2 Cutaneous lupus erythematosus (CLE)

CLE is an autoimmune disease that can present either as a primary dermatological disorder or as a manifestation within the spectrum of SLE. It is observed in 75%-85% of individuals diagnosed with lupus and distinguished by recurrent, photosensitive skin lesions that can give rise to scarring and alopecia (150). The pathogenesis of CLE is multifactorial, involving a complex interplay between genetic and environmental factors that lead to immune dysregulation. The mechanism remains incompletely understood (151).

Inflammation plays a key role in CLE, with the initiation of lesions likely rooted in a pro-inflammatory epidermis (152). Therefore, defining the cellular makeup in both lesional and nonlesional skin and characterizing key mediators of inflammatory changes are vital steps in identifying new therapeutic targets for CLE.

Type I interferons are central to lupus pathogenesis and may trigger disease onset in susceptible individuals (153). Anti-IFN strategies, including the anti-IFN-α monoclonal antibody sifalimumab and the IFN receptor-blocking anifrolumab, have been assessed in clinical trials, demonstrating acceptable safety profiles (154, 155). Recently, Billi et al. utilized scRNA-seq, Spatial-seq, and IMC to reveal that the normal-appearing skin of CLE patients exhibited a type I interferon-rich, prelesional microenvironment that affects gene transcription in all major skin cell types and disrupts cell-cell communication. Especially, lupus-enriched CD16+DCs underwent robust interferon education, acquiring proinflammatory phenotypes (90). In a subsequent study, Vazquez et al. discovered that discoid lupus erythematosus (DLE) and subacute cutaneous lupus erythematosus (SCLE) share similar skin immune microenvironments, and smoking might influence disease activity in CLE through neutrophils and endothelial GZMB. Notably, the findings suggest that pDCs are not the primary producers of IFN-1 in CLE (91). Nevertheless, pDCs may indeed play an important role in at least a subset of CLE patients. Litifilimab, a monoclonal antibody targeting BDCA2, is currently under investigation for CLE and has shown superiority over placebo in a Phase 2 trial (156). Further research is needed to ascertain the role of pDCs in lupus pathogenesis, especially for patients who respond to pDC targeting treatments.

Autoimmune responses and the involvement of B cells in the pathogenesis of SLE have been extensively documented (157). However, the role of skin-associated B cells in CLE is less evident. In a recent investigation, researchers found B cell gene expression signatures can help to distinguish CLE subtypes. Compared to ACLE and SCLE, DLE has a stronger B cell gene signature, particularly in patients with isolated cutaneous disease. This work lays the groundwork for future exploration into the potential utilization of a skin B cell score as a clinical marker for assessing SLE risk, particularly in DLE patients (92).

In addition, IMC was also used to identify baseline immunophenotypes that may predict the response to drug therapy. Patients were stratified based on their response to antimalarials: hydroxychloroquine (HCQ) responders, quinacrine (QC) responders, or nonresponders. HCQ responders had increased CD4+ T cells compared to QC responders. Nonresponders had decreased Treg cells compared to QC responders and increased central memory T cells compared to HCQ responders. QC responders expressed higher levels of pSTING and IFNκ, localized to conventional dendritic cells (cDCs), with the intensity correlating with cDC numbers. These findings reveal different immune cell compositions in CLE patients, guiding future research on precision medicine and treatment response (93).

While the current studies on CLE utilizing IMC have been constrained by small sample cohorts and a limited number of investigated markers, these endeavors vividly showcase the remarkable potential of this technology in studying phenotypic alterations of immune cells and their spatial associations within CLE skin tissue at high resolution.

#### 4.2.3 Systemic sclerosis (SSc)

SSc, commonly referred to as scleroderma, is an autoimmune disorder characterized by skin and internal organ fibrosis, as well as vasculopathy. The mortality rate in systemic sclerosis is notably high, particularly among individuals with diffuse cutaneous systemic sclerosis. Nonetheless, the mechanisms underlying the initiation of autoimmunity that ultimately leads to fibrosis, as well as the contribution of immune effector pathways to the pathogenesis of SSc, are still not fully understood (158, 159).

In a recent CyTOF study, notable disparities in T and B cell subset frequencies among SSc patients underscored disrupted immune architecture and the predominant presence of inflammatory senescent T cell modules (79). Examination of immune cell sorting revealed that mucosal-associated invariant T (MAIT) cells in SSc patients exhibited an activated phenotype, accompanied by increased expression of inhibitory molecules. MAIT cells constitute a subset of unconventional T cells characterized by an invariant TCR repertoire and high CD161 expression. Studies have demonstrated a reduction in MAIT cells in conditions such as Sjogren’s syndrome, RA, and SLE, showing their significant role in autoimmune disorders (160–163). Further employing high-dimensional techniques such as CyTOF, Phenocyler (formerly known as CODEX) to capture all MAIT cell subsets in SSc patients would be highly valuable.

Although microvascular alterations are the earliest histopathological manifestation of SSc, the vascular pathophysiology remains poorly understood. Notably, in SSc, infiltrating immune cells often accumulate in the perivascular area, and activated endothelial cells (ECs) emerge as a rich source of pro-fibrotic factors (164, 165). Moreover, ECs in SSc can undergo endothelial-to-mesenchymal transition (EndMT), acquiring a fibroblast-like phenotype (166). Recently, scientists utilized IMC to study vascular cell populations *in situ* and characterize their local vascular niche in SSc (95). They identified different subpopulations of vascular endothelial cells (VECs), lymphatic endothelial cells (LECs), and pericytes, uncovering an increased abundance of a novel endothelial cell cluster, CD34+;αSMA+ (alpha-smooth muscle actin); CD31+ VEC, in SSc. Interestingly, CD34+;αSMA+;CD31+ VECs express markers associated with EndMT, such as SNAI1, SNAI2, TWIST1, and ZEB1, and are located near immune cells and myofibroblasts. These VECs may represent an intermediate stage of endothelial cells transitioning to myofibroblasts through EndMT. Furthermore, the quantity of CD34+;αSMA+;CD31+ VECs correlates with clinical outcomes of progressive fibrotic remodeling, offering a novel cellular correlate for the interaction between vascular changes and fibrosis in SSc.

Researches into SSc fibrosis are ongoing, studies has revealed that cytokines released by activated inflammatory cells can induce fibroblasts-to-myofibroblasts transition and collagen release, playing a key role in fibroblast activation in SSc (167). Interleukin (IL)-1, IL-33, and IL-36 are potent pro-inflammatory cytokines recently discovered to be linked with the development of fibrotic tissue remodeling (168–170). The IL-1 Receptor Accessory Protein (IL1RAP) is an essential accessory receptor required for signaling through IL-1, IL-33, and IL-36 receptors, presenting potential as a target for simultaneously blocking all three cytokines (171). Using IMC, researchers revealed an increased protein levels in IL1RAP and related molecules in SSc skin compared to normal skin. *In vitro* treatment with anti-IL1RAP antibodies effectively blocked the response of human fibroblasts and endothelial cells to IL-1, IL-33, and IL-36. Moreover, therapeutic administration of the mouse anti-IL1RAP antibody mCAN10 showed promising anti-inflammatory and anti-fibrotic effects in mouse models of bleomycin-induced, cGvHD-induced, and topo-induced skin and lung fibrosis (94). These findings provide a rationale for targeted IL1RAP inhibition in SSc and have significant translational potential, as CAN10 has recently entered a phase I clinical trial and has been granted orphan drug designation for the treatment of SSc in the USA.

CyTOF/IMC is expected to continue providing new insights into the mechanisms of fibrotic tissue remodeling in SSc, thereby advancing the development of innovative therapeutic strategies for the disease.

#### 4.2.4 Hidradenitis suppurativa (HS)

HS, commonly referred to as acne inversa, is a debilitating chronic inflammatory skin disorder characterized by perifollicular lymphocytic infiltration and subsequent sebaceous gland loss. The disease involves the activation of both innate and adaptive immune cells, leading to an unrestrained and persistent immune response. Over time, this immune dysregulation results in intense pain, discharge of pus, irreversible tissue damage, and the formation of scars (172). A genetic predisposition, smoking, obesity and hormonal factors are established aetiological factors for HS (173). However, the immunopathogenesis of HS remains poorly understood.

A recent study used IMC and scRNA-seq to assess the infiltration and spatial localization of various immune cell subsets in HS skin lesions. The researchers found that B cells, plasma cells, immunoglobulin production, and complement activation are key factors in HS pathogenesis, with BTK and SYK pathways as central signaling networks (96). It’s worth noting that the BTK inhibitor (G-744) has been found to halt plasma cell generation in spontaneous and IFNα-accelerated NZB/W_F1 lupus models (174), while SYK has been shown to be pivotal in B cell antibody responses, memory B cell survival, and plasma cell proliferation (175, 176). These findings provide initial evidence for clinical trials targeting the BTK and SYK signaling pathways in HS.

Adalimumab (anti-TNFα) is the only FDA-approved drug for treating patients with HS, although approximately 30% of patients do not respond (177). Targeting a single cytokine may not suffice to treat HS, as numerous inflammatory pathways in both the skin and serum of HS patients are dysregulated. Targeting multiple dysregulated immune cells may yield better results. Through CyTOF analysis, Dimitrion et al. observed elevated CD38 expression on late NK cells, memory B cells, plasma blasts, pDCs, and I.Monos, which likely migrate to the skin to contribute to HS development (82). CD38 is primarily located in immune cells and serves multiple functions. It acts both as an enzyme, involved in NAD depletion and intracellular signaling, and as a receptor with adhesive properties (178). It has been reported that there is an increase in CD38 expression on peripheral immune cells in patients with SLE (76), while the anti-CD38 monoclonal antibody mezagitamab (TAK-079) has shown effectiveness in treating SLE refractory to anti-TNF (179). In summary, anti-CD38 immunotherapy may be a new management strategy for HS.

## 5 Conclusions and future perspectives

Over the last decade, multiplex single-cell proteomic technologies have emerged as a prominent focus in biomedical research. Progress in immune phenotyping technologies has ushered in an unparalleled level of cell subset resolution. CyTOF offers extensive multiplex single-cell analysis, enabling simultaneous measurement of cell surface markers and phosphorylation of orchestrators of biochemical responses. This capability aids in identifying biomarkers, novel pathways, therapuatic targets, and dysregulated cell types in immunological skin diseases. Conducting spatially-resolved single-cell analysis with IMC represents an exciting advancement in histological imaging platforms. The resulting high-dimensional histological images facilitate spatial analysis to identify distinct cell populations and cell–cell interactions central to cutaneous inflammation, and provide insights into activation/behavioral states based on skin tissue location. This capability will significantly enhance our understanding of dermatopathology. Additionally, CyTOF and IMC are being integrated with single-cell transcriptomics and whole-genome sequencing to enable unparalleled multi-omics analysis, facilitating a comprehensive exploration of disease mechanisms.

This review provides a detailed overview of mass cytometry and imaging mass cytometry in the context of investigating immune-related skin diseases. Integrating single-cell mass cytometry detection into basic and translational research could significantly enhance our understanding of disease subtyping, biomarkers, precision treatment, and prognosis prediction.

## Author contributions

MZ: Conceptualization, Writing – original draft. YC: Writing – review & editing. JG: Supervision, Writing – review & editing. FZ: Conceptualization, Writing – review & editing.
